# Circumpolar diversification of the *Ixodes uriae* tick virome

**DOI:** 10.1371/journal.ppat.1008759

**Published:** 2020-08-03

**Authors:** John H.-O. Pettersson, Patrik Ellström, Jiaxin Ling, Ingela Nilsson, Sven Bergström, Daniel González-Acuña, Björn Olsen, Edward C. Holmes

**Affiliations:** 1 Zoonosis Science Center, Department of Medical Biochemistry and Microbiology, Uppsala University, Uppsala, Sweden; 2 Marie Bashir Institute for Infectious Diseases and Biosecurity, School of Life and Environmental Sciences and School of Medical Sciences, The University of Sydney, Sydney, New South Wales, Australia; 3 Zoonosis Science Center, Department of Medical Sciences, Uppsala University, Uppsala, Sweden; 4 Department of Molecular Biology, Umeå University, Umeå, Sweden; 5 Laboratorio de Parásitos y Enfermedades de Fauna silvestre, Facultad de Ciencias Veterinarias, Universidad de Concepción, Chillán, Chile; University of Utah, UNITED STATES

## Abstract

Ticks (order: Ixodida) are a highly diverse and ecologically important group of ectoparasitic blood-feeding organisms. One such species, the seabird tick (*Ixodes uriae*), is widely distributed around the circumpolar regions of the northern and southern hemispheres. It has been suggested that *Ix*. *uriae* spread from the southern to the northern circumpolar region millions of years ago and has remained isolated in these regions ever since. Such a profound biographic subdivision provides a unique opportunity to determine whether viruses associated with ticks exhibit the same evolutionary patterns as their hosts. To test this, we collected *Ix*. *uriae* specimens near a Gentoo penguin (*Pygoscelis papua*) colony at Neko harbour, Antarctica, and from migratory birds—the Razorbill (*Alca torda*) and the Common murre (*Uria aalge*)—on Bonden island, northern Sweden. Through meta-transcriptomic next-generation sequencing we identified 16 RNA viruses, seven of which were novel. Notably, we detected the same species, Ronne virus, and two closely related species, Bonden virus and Piguzov virus, in both hemispheres indicating that there have been at least two cross-circumpolar dispersal events. Similarly, we identified viruses discovered previously in other locations several decades ago, including Gadgets Gully virus, Taggert virus and Okhotskiy virus. By identifying the same or closely related viruses in geographically disjunct sampling locations we provide evidence for virus dispersal within and between the circumpolar regions. In marked contrast, our phylogenetic analysis revealed no movement of the *Ix*. *uriae* tick hosts between the same locations. Combined, these data suggest that migratory birds are responsible for the movement of viruses at both local and global scales.

## Author summary

As host populations diverge, so may those microorganisms, including viruses, that are dependent on those hosts. To examine this key issue in host-microbe evolution we compared the co-phylogenies of the seabird tick, *Ixodes uriae*, and their RNA viruses sampled from the far northern and southern hemispheres. Despite the huge geographic distance between them, phylogeographic analysis reveals that the same and closely related viruses were found both within and between the northern and southern circumpolar regions, most likely reflecting transfer by virus-infected migratory birds. In contrast, genomic data suggested that the *Ix*. *uriae* populations were phylogenetically distinct between the northern and southern hemispheres. This work emphasises the importance of migratory birds and ticks as vectors and sources of virus dispersal and introduction at both the local and global scales.

## Introduction

Following the physical separation of a population into geographically isolated sub-populations (i.e. vicariance) genetic changes unique to each sub-population will accumulate. Given a sufficient period of time, such process may result in marked genetic separation. By combining phylogenetic and geographical information—that is, phylogeography [1,2]—it is possible to infer the spatial and evolutionary relationships among such subdivided populations. The analyses of these populations may include inferences on the direction of dispersal between subpopulations and if there have been multiple introductions into a particular geographic region. A recently colonised area is expected to exhibit less genetic diversity than the source population [1,2].

As host populations diverge, so will any microorganisms, including viruses, that are dependent on their hosts. Accordingly, analysis of genome sequence data from these microorganisms can provide additional, and sometimes more detailed, information about the evolutionary and epidemiological history of the host species [3,4]. Phylogenetic resolution of the patterns and processes of host-pathogen co-divergence is particularly strong in the case of RNA viruses in which mutational changes accumulate far more rapidly than in their hosts [4]. For example, analysis of the phylogeny of feline immunodeficiency virus (FIV) provided important information on the recent population and demographic history of its feline host, the cougar *Puma concolor*, that was not apparent in host genetic data [5].

Ticks (order Ixodida) are among the most diverse groups of ectoparasites. There are close to 900 species of both soft- and hard-bodied ticks within the Ixodida [6,7], of which the genus *Ixodes* is the most species rich group with nearly 250 species [6,8–10]. Within this genus, *Ix*. *uriae* is the only known tick with a circumpolar distribution in both the northern and southern hemispheres. This species parasitizes close to 100 different vertebrate species, the majority of which are seabirds that breed in dense colonies [11]. In the northern hemisphere, the most commonly recorded hosts are birds of the order Charadriiformes, mainly *Alcidae* and *Laridae*, and in the southern hemisphere they are mainly species of the Spheniciformes and Procellariiformes [11–14]. Like most hard ticks, *Ix*. *uriae* has three active life-stages (larva, nymph and adult) whose questing behaviour is most prevalent during the summer months with a peak during June–July in the northern hemisphere and December–January in the southern hemisphere [15–18]. Each active stage takes a single blood-meal from a host during 3–12 days depending on the tick’s life-stage. The duration of the life cycle depends on environmental temperatures and may last from three to seven years, among the longest seen in ticks [13,15,19,20]. Importantly, and perhaps as a consequence of its host and habitat adaptation, *Ix*. *uriae* can tolerate temperatures as low as -30°C and as high as +40°C [21], forming aggregations in moist rocky microhabitats [17,21]. *Ix*. *uriae* is a well-known vector of multiple different viruses and bacteria, including *Borrelia burgdorferi* sensu lato [22,23], the agent of Lyme disease, and Gadgets Gully virus [24,25] amongst others (reviewed in [11]). However, although some viruses and bacteria may have zoonotic potential and humans are bitten by *Ix*. *uriae*, there is limited evidence for human disease associated with *Ix*. *uriae* transmission [11].

Data from both mitochondrial and nuclear genes suggest that *Ix*. *uriae* diverged from its most recent common ancestor, *Ix*. *holocyclus*, approximately 91 million years ago, and that the *Ix*. *uriae* species complex shared a common ancestor some 22 million years ago [26]. Subsequently, *Ix*. *uriae* was introduced, possibly twice, into the northern hemisphere from the likely ancestral Australasian population approximately 10 million years ago [26]. Thereafter, both the southern and northern populations diversified into geographically structured subpopulations with no evidence of dispersal between them [26]. However, as some birds can migrate long distances, it is important to determine whether there has been any recent viral dispersal between the two polar regions and if there is any gene flow between the two *Ix*. *uriae* sub-populations. Here, by comparing the viromes of *Ix*. *uriae* collected from seabirds–the Common murres’ (*Uria aalge*) and Razorbills’ (*Alca torda*)–from the northern hemisphere and from around a penguin (*Pygoscelis papua*) colony in the southern hemisphere, we investigated whether there has been virus dispersal either within or between the northern and southern hemispheres.

## Results

**Fig 1:**
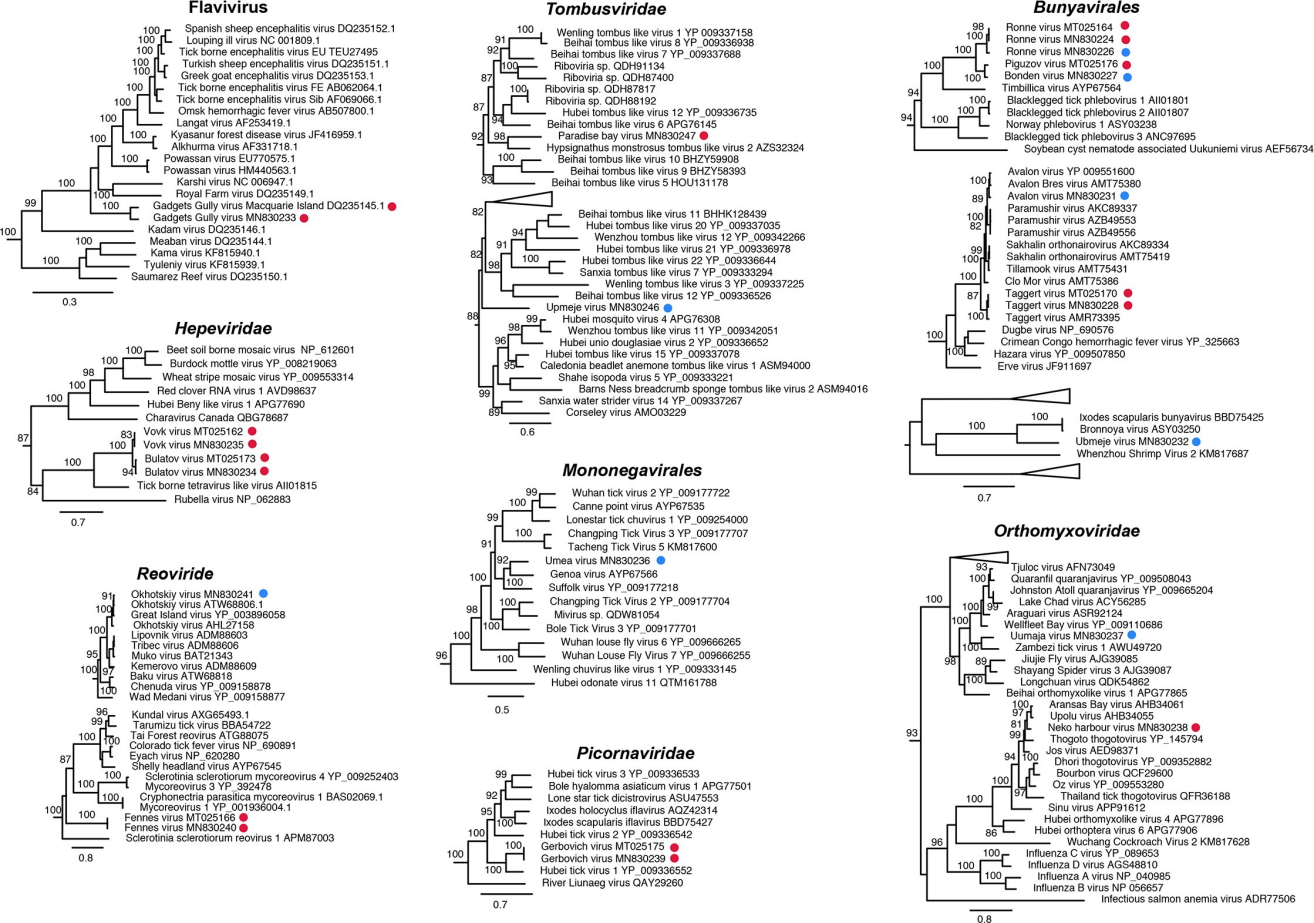
Phylogenetic analysis of all the viruses identified here within their respective virus groups, including representative publicly available viruses. Viruses identified in the current and a related study (27) are indicated, with red and blue circles for viruses identified from the southern and northern circumpolar regions, respectively. Numbers on branches indicate Shimodaira–Hasegawa (SH) support values (only branches with SH support ≥80% are indicated) and branch lengths are scaled according to the number of amino acid substitutions per site. All phylogenetic trees were mid-point rooted for clarity only.

In total, we generated 16 RNA sequencing libraries from 33 ticks, all of which were engorged adult female *Ix*. *uriae* individuals: 10 libraries using two tick individuals from the southern hemisphere, and six libraries from the northern hemisphere comprising five with two tick individuals and one with three tick individuals (S1 Table). These libraries were sequenced to a high depth and assembled *de novo*. Across the libraries as a whole we identified 16 RNA viruses, seven of which were novel based on RNA-dependent RNA polymerase (RdRp) sequence similarity. The viruses identified belong to the following orders/families: *Bunyavirales* (N = 5), *Hepeviridae* (N = 2), *Flavivirus* (genus) (N = 1), *Mononegavirales* (N = 1), *Orthomyxoviridae* (N = 2), *Picornaviridae* (N = 1), *Reoviridae* (N = 2) and *Tombusviridae* (N = 2) (Fig 1).

The individual viruses discovered were at abundance levels ranging from 3 to 10,203 reads per million and the total viral abundance per library, approximated from all virus RdRp reads mapped in positive libraries, varied between 14–12,257 reads per million. Correspondingly, the abundance of *Ix*. *uriae*, approximated via the host COX1 gene, varied between 1,359–8,101 reads per million (Table 1). Four viruses—Gadgets Gully virus (*Flavivirus*), Taggert virus (*Bunyavirales*), Neko harbour virus (*Orthomyxoviridae*) and Upmeje virus (*Tombusviridae*)—were highly abundant (i.e. abundance levels above 1,000 reads per million or more than 0.1% of the total number of reads per library). Indeed, Gadgets Gully virus, Taggert virus and Upmeje virus were more abundant than the host COX1 gene (Table 1), reaching 1,704, 1,768 and 10,203 reads per million, respectively, suggesting that they are tick-associated viruses. However, any definitive tick-association of these viruses will need to be determined in future studies.

**Table 1:** Presence and estimates of virus abundance across libraries.

	*Ixodes uriae*, Antarctica										*Ixodes uria*e, northern Sweden						
Virus name (abbreviation)	IU1	IU2	IU3	IU4	IU5	IU6	IU7	IU8	IU9	IU10	IU15	IU16	IU17	IU18	IU19	IU20	IH 0.1%
Ronne virus (RONV)	14	24	19	16	21	22	4	0	4	7	1	3	6	1	1	1	1
Ronne virus (RONV)	1	3	2	2	2	2	1	0	0	1	10	24	69	7	9	3	3
Bonden virus (BONV)	0	1	0	0	1	0	1	0	0	0	7	13	45	3	4	1	2
Taggert virus (TAGV)	1	4	1	5	1	971	**2175**	0	**1768**	1	5	1	1	1	1	1	103
Avalon virus (AVAV)	0	0	0	1	0	11	25	0	21	0	312	0	0	0	0	0	15
Ubmeje virus (UBEV	0	0	0	0	0	0	0	0	0	0	17	203	1	0	0	41	13
Gadgets Gully virus (GGYV)	0	0	0	3	0	0	0	0	**1704**	0	0	0	1	0	0	0	75
Bulatov virus (BULV)	0	70	88	101	39	0	35	8	12	92	0	0	0	0	0	0	5
Vovk virus (VOVV)	0	14	13	136	24	0	236	3	90	11	0	0	0	0	0	0	4
Umea virus (UMEV)	1	0	1	0	0	1	0	0	0	1	73	506	**1438**	135	92	15	66
Uumaja virus (UUMV)	1	0	0	1	0	0	0	0	0	1	59	233	502	44	54	80	23
Neke harbour virus (NEHV)	0	0	1	0	**2008**	0	0	0	2	85	0	1	0	0	0	1	95
Gerbovich virus (GERV)	0	369	3	0	552	0	0	0	3	0	0	0	0	0	0	0	26
Fennes virus (FENV)	0	0	12	61	0	14	0	0	172	0	0	0	0	0	0	0	8
Okhotskiy virus (OKHV)	0	0	0	0	0	0	0	0	0	0	0	296	0	0	0	0	19
Upmeje virus (UPEV)	3	2	2	2	2	1	1	0	2	1	2	2	**10203**	2	573	661	469
Paradise bay virus (PABV)	4	0	0	0	0	0	0	115	0	0	0	0	0	0	0	0	48
*Ix*. *uriae* COX1	1548	2555	2967	1359	3520	2824	3747	2598	1505	1807	8101	4678	1964	3179	4819	4580	
Total virus***	14	476	132	315	2645	1006	2449	1231	3750	110	478	1275	12257	189	732	765	

Abundance values are expressed as reads per million (see Materials and Methods)

IH 0.1% is the assumed index-hopping cut-off in relation to the most abundant library.

* Values in bold indicate libraries with an abundance greater than 1000 reads per million.

** Libraries in bold within a square indicate abundance levels greater than that of the host.

*** Total virus abundance for a single library as reads per million.

### Circumpolar virome comparison

Of the 16 viruses identified, nine were found in *Ix*. *uriae* ticks sampled from a Gentoo penguin (*Pygoscelis papua*) colony at Neko harbour, Antarctica, and seven were found from *Ix*. *uriae* ticks collected from Razorbill (*Alca torda*) and Common murre (*Uria aalge*) seabirds on Bonden island in the Gulf of Bothnia, northern Sweden (Fig 1, Table 1, S1 Table). The geographical separation of the different virus species’ was confirmed when there was no overlap of individual virus contigs between the two sampling sites (i.e. a particular virus or virus variant was only found in the northern or southern hemisphere sampling site, but not both) (Table 1). This analysis revealed that the majority of viruses were found either in the northern or southern hemisphere *Ix*. *uriae* sequence libraries, but not both (Table 1, S2 Table). Notably, however, we identified two variants of Ronne virus (*Bunyavirales*) in both Antarctica and northern Sweden (Fig 1, Table 1). Ronne virus has been previously identified from ticks in Antarctica [27]. One variant of Ronne virus identified here, also sampled from Antarctica, was highly similar (99.9% amino acid similarity; 99.7% nucleotide similarity) to that previously identified, whereas the second variant, recovered from ticks collected from the north of Sweden, was more divergent in sequence (93.2% amino acid similarity; 80.3% nucleotide similarity). Such genomic similarity is indicative of a relatively recent dispersal event between the northern and southern tick populations, although the direction of migration remains to be determined. Similarly, we identified a novel bunyavirus, Bonden virus, from northern Sweden that is the closest known relative (90.3% amino acid similarity; 78.8% nucleotide similarity) of Piguzov virus identified from *Ix*. *uriae* in Antarctica (Fig 1) [27]. Although it is unclear when these two viruses diverged, they clearly point to an historical dispersal event between the two poles.

It was also notable that we identified both Taggert virus (*Bunyaviridae*) and Gadgets Gully virus (*Flaviviridae*) from the Neko harbour sampling site. Both these viruses have previously been found on Macquarie island, south-east of the Australian continent [24,25,28]. From northern Sweden we identified Avalon virus (*Bunyaviridae*) and Okhotskiy virus (*Reoviridae*), previously isolated from *Ix*. *uriae* from the Great island, Newfoundland, Canada, and from islands around the sea of Okhotsk, respectively [29–31]. The finding of Avalon virus, Gadgets Gully virus, Okhotskiy virus and Taggert virus at these new locations again indicates that there has been widespread geographical dispersal of viruses within each hemisphere. Although there was some tentative signal for the presence of Avalon virus in two tick libraries from the southern sampling site (dark grey cells, Table 1), these likely represent false-positives due to incorrect genomic mapping as some sequence motifs are similar to those in Taggert virus that is highly abundant in these libraries.

Less clear is whether the divergence of these variants justifies their classification as new virus species. For example, the variant of Gadgets Gully virus identified here shares 92.7% amino acid similarity and 80.7% nucleotide similarity to the currently available genome (YP_009345034.1) originally isolated in 1976 [24]. Given the commonly applied rules for species delineation (< 90% amino acid similarity and/or < 80% nucleotide similarity), the Gadgets Gully variant detected here is on the cusp of being considered a new species. In addition, as the original variant of Gadgets Gully virus(CSIRO122) was isolated in 1976 from *Ix*. *uriae* collected from Macquarie island, more than 40 years before those identified here, it is likely that this virus has circulated in this geographic for several decades. Similarly, one of the original variants of Okhotskiy virus isolated in 1972 (ATW68806.1) and that sequenced here share 97.7% amino acid and 87.6% nucleotide similarity in the VP1 (RdRp) segment, also compatible with the idea that this virus has circulated *in situ* for more than 40 years. Regardless of how and when these viruses transferred to new locations, it is evident that there has been circumpolar dispersal of *Ix*. *uriae* associated viruses.

### Virus–host co-evolution and migration

**Fig 2:**
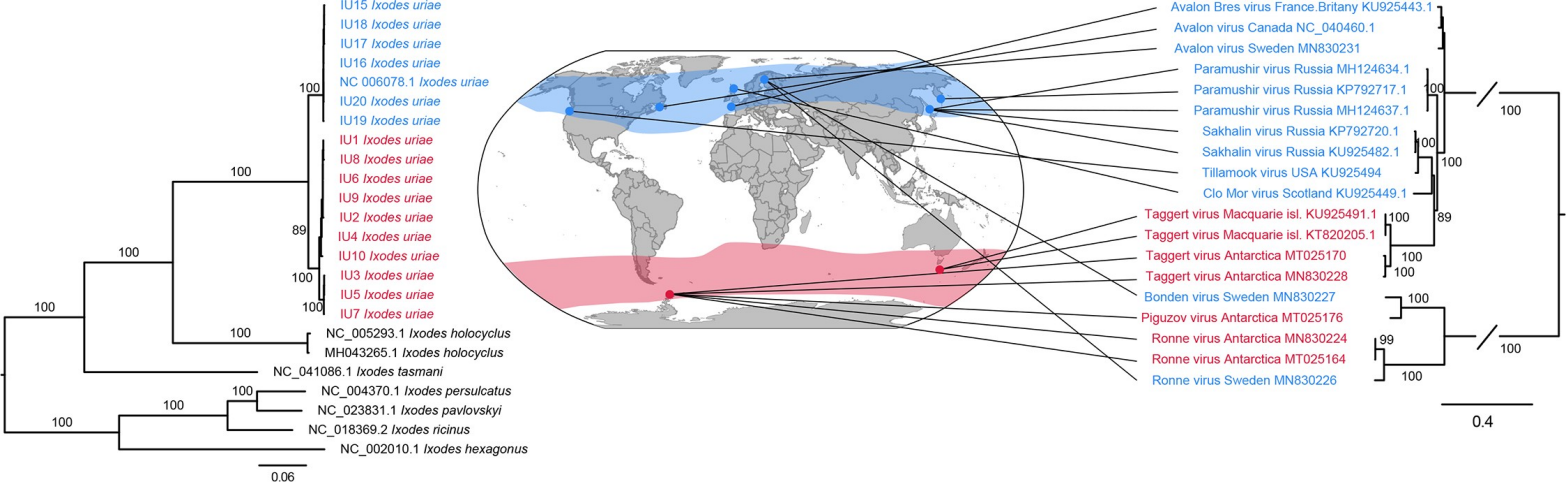
Phylogenetic analysis of near complete host mitochondrial genome data (mined from each library) and of closely related bunyaviruses found at either the southern (red colour) or northern (blue colour) circumpolar sampling sites. Lines connect viruses with their sampling locations. Numbers on branches indicate SH support values (only branches with SH support ≥80% are indicated) and branch lengths are scaled according to the number of amino acid substitutions per site. All phylogenetic trees were midpoint-rooted for clarity only.

To better understand virus–tick co-evolution, the host mitochondrial genome of *Ix*. *uriae* was mined from all sequence libraries, and a phylogenetic analysis performed on two sets of representative outgroup and ingroup ixodid species: one utilising complete mitochondrial genome (Fig 2) and a second comprising all mitochondrial gene sequences regardless of length (S1 Fig). For comparison, we performed a phylogenetic analysis of RdRp sequences from a sub-set of closely related bunyaviruses found at either the northern or the southern sampling sites. Although our *Ix*. *uriae* sequences only represent a small portion of the distributional range of both circumpolar regions that this species inhabits, the results obtained are in agreement with those of previous studies in identifying two distinct tick populations with no evidence of dispersal between the northern and southern hemispheres (Fig 2, S1 Fig) [26,32,33]. In addition, we observed longer branch lengths in the Antarctic *Ix*. *uriae* mitochondrial sequences than those from northern Sweden, and the average number of nucleotide differences per site (π) was greater in the southern (complete genomes [mean and SE]: 44.04 ± 3.51; all mitochondrial data [mean and SE]: 18.57 ± 2.77) than the northern population (complete genomes [mean and SE]: 12.95 ± 2.18; all mitochondrial data [mean and SE]: 4.51 ± 1.08). Although more data is clearly needed, these patterns of genetic diversity are compatible with the idea that the southern circumpolar population represents the ancestral population.

The phylogenetic analysis of a subset of closely related bunyaviruses reveals several patterns of geographic dispersal (Fig 2). The clade that includes Avalon virus, Paramushir virus and Taggert virus suggests that *Ix*. *uriae*-associated viruses moves within the circumpolar regions inhabited by *Ix*. *uriae*. For example, Avalon virus, closely related to Paramushir virus [34], has been found at the Great island, Newfoundland, Canada [29], along the coast of Brittany, France [35], and now in the northern part of Sweden. As noted earlier, there are also indications of movement between the northern and southern circumpolar regions. Indeed, the close phylogenetic relationships of both Bonden virus and Ronne virus to southern circumpolar viruses clearly demonstrate that viruses are able to move between these two highly disjunct regions. More generally, that there are several different bird species, many of which are known hosts for *Ix*. *uriae* [11], that have migratory routes within and between the circumpolar regions [36] and cover the geographical distribution of *Ix*. *uriae*, suggests that *Ix*. *uriae* associated viruses are being transported by migratory birds within and between the circumpolar regions. Hence, it is possible that these viruses may be present in all regions with permanent populations of *Ix*. *uriae*.

### Novel and previously identified RNA viruses

We identified a number of other novel and previously identified RNA viruses. For example, Bulatov virus and Vovk virus (*Hepeviridae*) have previously been identified in Antarctic ticks [27] and were also discovered here (Fig 1, Table 1). They share a common ancestor and are relatively similar genetically (86.5% amino acid identity), but are in themselves divergent, sharing only 35.1% and 36.8% amino acid similarity, respectively, to the tick-borne tetravirus like virus (AII01815). Umea virus (*Mononegavirales*) was discovered in several of the tick libraries from northern Sweden and was relatively abundant (1,438 reads per million) in one library (Table 1). It shares a most recent common ancestor with Genoa virus (Fig 1) identified from *Ix*. *holocyclus* ticks from Australia [37], but is again relatively divergent (56.4% amino acid similarity). Similarly, Ubmeje virus (*Bunyavirales*) was identified in several libraries from northern Sweden, although it was not abundant (Table 1). Ubmeje virus shared only ~35.8% and 35.9% amino acid identity to Bronnoya virus and Ixodes scapularis bunyavirus, both previously observed in ixodid ticks [38,39], and which together form a monophyletic group (Fig 1).

In the Antarctic sequence libraries we observed Gerbovich virus (*Picornaviridae*) that has previously been described in this region [27]. The two variants are very similar in sequence (99.7% amino acid similarity, 99.3% nucleotide similarity), but have only 56.5% amino acid similarity with their closest relative, Hubei tick virus 1, identified a pool of ticks from China (Fig 1) [40]. Aside from Okhotskiy virus (*Reoviridae*) described above, we also identified Fennes virus in the Antarctic sequence libraries, with near identical sequence similarity (99.8% amino acid similarity, 100% nucleotide similarity) to the sequence of this virus identified previously [27]. Fennes virus represents a highly divergent lineage, sharing only 31.0% amino acid similarity with Shelly headland virus discovered in *Ix*. *holocyclus* ticks from Australia [37].

Two novel orthomyxoviruses were identified: Uumaja virus from northern Sweden and Neko harbour virus from Antarctica (Fig 1, Table 1). Uumaja virus is most closely related to Zambezi tick virus 1, identified in a *Rhipicpehalus* sp. tick collected in Mozambique [41]. Neko harbour virus was found to be abundant (more than 2,000 reads per million) in a single library (Table 1) and clusters with Aransas Bay virus [42], to which it was most similar (82.7% amino acid similarity), as well as Jos virus [43], Thogoto thogotovirus [44] and Upolu virus [42]. All these viruses are tick-derived and originate from different continents. This pattern is indicative of a long-term association between ticks and these viruses.

Finally, we identified two novel and divergent viruses within the *Tombusviridae*: Paradise bay virus from Antarctica and Upmeje virus from the northern Sweden (Fig 1, Table 1). Paradise bay virus grouped with Hypsignathus monstrosus tombus-like virus 2 (50.9% amino acid similarity), a virus sequenced from blood samples of Hammer-headed fruit bats (*Hypsignathus monstrosus*) collected in the Republic of the Congo [45]. Upmeje virus was found to be highly divergent, sharing only 35.2% amino acid similarity with Sanxia water strider virus 14 and did obviously cluster with any other virus (Fig 1). It is noteworthy that Upmeje virus was the most abundant in our study, attaining more than 10,000 reads per million, some five times greater than the host marker gene abundance in the same library (Table 1).

## Discussion

Ticks are the most important blood-feeding arthropods in temperate and polar regions [46–54]. Their ability to adapt to harsh, climatologically variable environments and a wide array of vertebrate hosts has enabled them to become established in many habitats and geographic locations. In addition, ticks are well-known vectors of multiple viruses, including those that are known pathogens of humans and other animals, as well as viruses considered or likely to be symbionts [37,38,55]. We studied the virome of the seabird tick, *Ix*. *uriae*, that is distributed across both circumpolar regions, as a means to understand virome composition, host–virus co-divergence and the long-distance dispersal of tick-borne viruses. In particular, we assessed whether we could infer dispersal events within and between the southern and northern circumpolar regions that are separated by a substantial geographic distances.

Overall, we identified 16 RNA viruses of which seven were novel. Of the nine viruses previously discovered, several have previously been documented in *Ix*. *uriae* and some have been shown to have pathogenic properties to either birds and/or humans [56,57]. For example, Gadgets Gully virus was originally found on Macquarie island, south-west of New Zealand, in the 1970s [24,58], again from ticks collected there in 2002 [25], and now in ticks collected in 2018 at Neko harbour, Antarctica. The level of diversity between those sequences determined previously and that in the present study suggests that Gadgets Gully virus has been maintained in circumpolar regions for several decades, if not centuries. Similarly, we identified Avalon virus, previously found in Canada and France, in the ticks collected at Bonden island, Sweden. This supports the idea that viruses are being transported within the circumpolar regions inhabited by *Ix*. *uriae*. These viruses could either be transported with the ticks carried by seabirds during migration or directly by infected birds. Indeed, several well-known bird-hosts of *Ix*. *uriae* have circumpolar migration patterns. For example, in the southern ocean, birds of the order Procellariiformes show circumpolar migration involving many stopover sites [59,60]. In the arctic region, many Charadriiform birds undertake seasonal long distance longitudinal migrations. Similarly, the Black legged kittiwake (*Rissa tridactyla*), Atlantic puffin (*Fratercula arctica*) and Thick billed murre (*Uria lomvia*) all show seasonal movements between the eastern and western north Atlantic [61,62].

We also saw evidence of historic movement of viruses between the northern and southern circumpolar regions. In particular, our phylogenetic analysis revealed that Bonden virus, identified in ticks from northern Sweden, was closely related to Piguzov virus from Antarctica [27]. Similarly, Ronne virus, present in Antarctica, was also found in northern Sweden (Figs 1 and 2). The close evolutionary relationship of viruses from the northern and southern circumpolar regions suggests that they have moved between the poles after the *Ix*. *uriae* population diverged into two sub-populations. In addition, as the northern and southern *Ix*. *uriae* populations are phylogenetically distinct [26], it seems likely that it is viruses rather than the ticks that are transferred between the two polar regions. This, in turn, implies that it is virus-infected migratory birds that transport viruses between the poles. Although some birds species migrate very long distances, few are known to move between the Arctic and Antarctic regions. The Arctic tern (*Sterna paradisaea*) performs the longest migration of any avian species, traveling the distance between Antarctica to southern Greenland, approximately 24,000 km, in around 40 days [63]. Although there are no records of *Ix*. *uriae* on this species, it is likely that they can become infested as they breed in dense colonies near bird species that are well-known hosts of *Ix*. *uriae*. As noted above, Procellariiform birds are long distance migrators and a study using geolocators of the Short-tailed shearwater (*Ardenna tenuirostris*) revealed that this species migrated to south of the Antarctic Polar Front after their breeding period in Tasmania, and following a stopover migrated northward to spend the Arctic summer in a location as far north as the Bering sea [64]. Although the Short-tailed shearwater can migrate the distance in as little as 11–16 days, it remains almost exclusively at sea, only touching land during the breeding season. Hence, tick dispersal by this species seems unlikely. The Sooty shearwater (*Ardenna grisea*) also undertakes long distance trans-equatorial migrations [65], and other Procellariiform birds breeding in the Antarctic region, such as the Black-browed albatross (*Thalassarche melanophris*) and the South polar skua (*Stercorarius maccormicki*), have been occasionally observed in the Arctic region. As both the nymph and adult tick can feed for up to 12 days [13,66], these birds could theoretically act as vehicles for inter-polar virus spread. Hence, although the *Ix*. *uriae* phylogeny suggests that there has been no movement of ticks among the southern and northern circumpolar regions, it cannot be excluded that there are as yet unsampled locations in either region where population admixture could occur.

Under what circumstances could a tick then be transferred between the two polar-regions? Unless a journey is made directly between the polar-regions, which is theoretically possible in the case of Short-tailed shearwater, it would be necessary to occur sequentially both with respect to bird stop-over and tick life-stage development. For example, a nymph would initially latch onto the host shortly prior the bird migration, feed for the entire duration of the flight, and develop to the next life-stage during the stop-over. The adult tick would then need to find a new host to continue its journey. Given the long distances and time during which tick and virus have to survive, such events are unlikely to take place in one migration step. At the same time, that we could identify two clear cases of cross-circumpolar dispersal of closely related viruses from such a small sample of viruses indicates that cross-circumpolar transmission may not be infrequent.

The finding of several previously discovered and novel viruses that are known or likely to be tick-associated raises interesting questions about how these viruses are maintained in nature. In the northern circumpolar distribution the tick life cycle can last up to seven years depending on host availability and temperature, and ticks may spend up to eleven months of the year off the host in aggregations formed in moist environments [13,15,17].For viruses to be maintained and transmitted yearly within the tick population, it is arguable that unless the bird hosts develop a chronic viral infection that lasts for a year, or that these viruses are transmitted via other routes than via blood (i.e. directly between hosts), the maintenance of these viruses at a particular location is to a large extent driven by tick behaviour and presence. For example, in the case of tick-borne encephalitis virus, it is hypothesised that *Ix*. *ricinus* acts as both the virus reservoir and vector [67,68]. In particular, the behaviour of *Ix*. *uriae* forming off-host aggregations, combined with the occasionally relatively short questing period for specific hosts [15,19,69], suggests that the tick non-viraemic co-feeding and trans-stadial transmission [49,67,70,71] of viruses are important mechanisms for the establishment and maintenance of viruses in circumpolar environments. However, despite the key role played by ticks, our study suggests that it is the avian host that likely functions as the dispersal agent of viruses among the circumpolar regions.

In sum, we have shown that the seabird tick *Ix*. *uriae* harbours an extensive diversity of viruses belonging to several different families and orders of RNA viruses, and that there has been a transfer of viruses both within and between the northern and the southern circumpolar regions. As such, we stress the importance of the millions of birds that each year migrate across the globe and that have the capacity to transfer viruses to and from adjacent and distant geographical areas.

## Materials and methods

### Tick collection and total RNA extraction

Adult female *Ix*. *uriae* ticks were collected during 2016–2017 from the Bonden island bird station in northern Sweden (lat/long: 63.433617, 20.038344) and from the ground around a Gentoo penguin (*Pygoscelis papua*) colony at Neko harbour, Antarctica (lat/long: -64.824066, -62.665999), during 2018. All ticks were morphologically keyed to species [72] and were subsequently stored in -80°C until further processing. Prior to total RNA extraction, ticks were washed in PBS buffer two times and then pooled (S1 Table). Total RNA from 16 tick pools was then extracted using the RNeasy Plus Universal kit (Qiagen) following the manufacturer’s instructions.

### Sequence library construction and sequencing

Sequencing libraries, data generation and analysis was performed as previously described [38,73]. Briefly, ribosomal RNA (rRNA) was depleted from the total RNA extracts using the Ribo-Zero Gold (human-mouse-rat) kit (Illumina) following the manufacturer’s instructions. RNA sequencing libraries were then prepared for all rRNA depleted extracts using the TruSeq total RNA library preparation protocol (Illumina) followed by paired-end (150 bp read-length) sequencing on a single Illumina HiSeq X10 lane by the Beijing Genomics Institute, Hong Kong. The raw sequence output was then quality trimmed with Trimmomatic v.0.36 [74] using the default settings for paired-end sequence data and assembled *de novo* using Trinity v.2.5.4 [75] with read normalisation apart from default options.

### Virome analysis and presence across libraries

All *de novo* assembled contigs were initially screened against the complete non-redundant nucleotide and protein databases (NCBI GenBank) using blastn v.2.6.0+ [76] and Diamond v.0.9.15.116 [77], respectively, employing cut-off e-values of 1 × 10^−5^ for both methods. To further assess the data and to identify potential endogenous viral elements, all assemblies indicative of RNA virus origin were screened using the NCBI Conserved Domain Database (www.ncbi.nlm.nih.gov/Structure/cdd/cdd.shtml) with an expected value threshold of 1 × 10^−3^. Relative abundance of the identified viruses was determined by comparing the mapping results of the *Ix*. *uriae* mitochondrial cytochrome C oxidase I (COX1) gene (NC_006078.1, positions 1214–2758) against all RdRp containing contigs using Bowtie2 v.2.3.4 [78], employing the default local setting for all libraries. Relative abundance was calculated as reads per million: that is, the number of reads mapped to a contig divided by the total number of reads in a library multiplied by a million. A particular virus was considered abundant if (i) it represented >0.1% of total ribosomal RNA depleted reads in the library, equivalent to a reads per million value of 1000 or more, and (ii) if the abundance was higher than that of the host COX1 gene [38,73]. If the relative abundance of a virus contig was less than 1 read per million mapped, or below the level of cross-library contamination due to index-hopping, assumed here as 0.1% of the most abundant library for the virus in question, the library was considered negative for the presence of the virus contig. A virus was considered novel if the RdRp region showed < 90% amino acid or < 80% nucleotide similarity to any previously identified virus.

### Virus evolutionary history

To infer the evolutionary history of all the RNA viruses identified here they were combined with representative amino acid data sets of the RNA-dependent RNA-polymerase genes of viruses from the orders *Bunyavirales*, *Mononegavirales*, *Orthomyxovirales*, the families *Picornaviridae*, *Reoviridae*, the Hepe-Nido-like and Tombus-Noda-like groups, and the genus *Flavivirus*. These sequences were then aligned using the E-INS-i algorithm in Mafft v.7.271 [79]. To reduce alignment uncertainty, regions that aligned poorly were removed using TrimAl v. v1.4.rev15 [80] under the ‘strict’ settings. Each alignment was then subjected to model testing to determine the best-fit model of amino acid substitution using ModelFinder [81] via IQ-TREE v.1.6.12 [82]. Finally, maximum likelihood phylogenetic trees of each data set were estimated using the IQ-TREE package, implementing a stochastic hill-climbing nearest-neighbour interchange tree search. Phylogenetic robustness was assessed using Shimodaira–Hasegawa (SH)-like branch supports.

### Virus–tick evolutionary history

To compare the evolutionary history of *Ix*. *uriae* and a subset of closely related *Bunyavirales* found at both sampling sites (see Results), the mitochondrial genome of *Ix*. *uriae* (NC_006078.1) was used as reference for mapping with Bowtie2 v.2.3.4 [78], with local settings, against all sequence libraries. Two alignments were constructed for the mitochondrial nucleotide consensus sequences present in each library: (i) only complete *Ixodidae* reference genomes (N = 24), and (ii) complete genomes of *Ix uriae* and *Ix holocyclus* (N = 19) from the complete genome alignment combined with shorter mitochondrial gene sequences (N = 148) taken from two previously published data sets [26,33]. Both alignments were constructed using the G-INS-i algorithm in Mafft v.7.271 [79]. Following visual inspection of the alignment in AliView v.1.26 [83], model testing and estimation of a maximum likelihood phylogenetic tree was performed in IQ-TREE as described above. The corresponding virus phylogeny was inferred using the nucleotide RdRp open reading frame sequences of a subset of bunyaviruses, keeping the open reading frame intact and utilising the same model testing and phylogenetic tree inference procedure as described above. All phylogenetic trees computed were visually compared and edited with FigTree v.1.4.3 (https://github.com/rambaut/figtree/). Comparison of genetic diversity between the northern and southern populations was undertaken by computing the number of base differences per site averaged over all sequence pairs between the two populations (i.e. π) using Mega X v.10.1.1 [84].

## Supporting information

S1 Table Sequence library information regarding sampling location, host species and collection year. (XLSX)

S2 Table Read mapping results for each identified virus and host gene per individual library. (XLSX)

S1 Fig Phylogenetic analysis of *Ixodes uriae* based on complete mitochondrial genomes and with the addition of shorter mitochondrial gene sequences. (PDF)

## References

[1] Avise JC. Phylogeography: the history and formation of species. Cambridge, Mass: Harvard University Press; 2000.

[2] Avise JC, Bowen BW, Ayala FJ. In the light of evolution X: Comparative phylogeography. Proc Natl Acad Sci USA. 2016;113: 7957–7961. 10.1073/pnas.1604338113 27432955 PMC4961136

[3] Whiteman NK, Parker PG. Using parasites to infer host population history: a new rationale for parasite conservation. Anim Conserv. 2005;8: 175–181. 10.1017/S1367943005001915

[4] Nieberding CM, Olivieri I. Parasites: proxies for host genealogy and ecology? Trends Ecol Evol (Amst). 2007;22: 156–165. 10.1016/j.tree.2006.11.012 17157954

[5] Biek R, Drummond AJ, Poss M. A virus reveals population structure and recent demographic history of its carnivore host. Science. 2006;311: 538–541. 10.1126/science.1121360 16439664

[6] Nava S, Guglielmone AA, Mangold AJ. An overview of systematics and evolution of ticks. Front Biosci (Landmark Ed). 2009;14: 2857–2877. 10.2741/3418 19273240

[7] Sonenshine DE, Roe RM, editors. Biology of ticks. 2nd ed New York: Oxford University Press; 2014.

[8] Oliver JH. Biology and Systematics of Ticks (Acari:Ixodida). Annu Rev Ecol Syst. 1989;20: 397–430. 10.1146/annurev.es.20.110189.002145

[9] Barker SC, Murrell A. Systematics and evolution of ticks with a list of valid genus and species names. Parasitology. 2004;129 Suppl: S15–36. 10.1017/s0031182004005207 15938503

[10] Guglielmone AA, Robbins RG, Apanaskevich DA, Petney TN, Estrada-Peña A, Horak I, editors. The hard ticks of the world: Acari: Ixodida: Ixodidae. Dordrecht: Springer; 2014.

[11] Muñoz-Leal S, González-Acuña D. The tick Ixodes uriae (Acari: Ixodidae): Hosts, geographical distribution, and vector roles. Ticks Tick Borne Dis. 2015;6: 843–868. 10.1016/j.ttbdis.2015.07.014 26249749

[12] Wilson N. Acarina: Metastigmata: Ixodidae of South Georgia, Heard and Kerguelen. Pac Insects Monogr. 1970;23: 78–88.

[13] Eveleigh ES, Threlfall W. The biology of Ixodes (Ceratixodes) uriae White, 1852 in Newfoundland. Acarologia. 1975;16: 621–635. 1179956

[14] McCoy KD, Boulinier T, Schjørring S, Michalakis Y. Local adaptation of the ectoparasite Ixodes uriae to its seabird host. Evol Ecol Res. 2002;4: 441–456.

[15] Barton TR, Harris MP, Wanless S, Elston DA. The activity periods and life-cycle of the tick Ixodes uriae (Acari: Ixodidae) in relation to host breeding strategies. Parasitology. 1996;112 (Pt 6): 571–580. 10.1017/s0031182000066154 8684831

[16] Mangin S, Gauthier-Clerc M, Frenot Y, Gendner J-P, Le Maho Y. Ticks Ixodes uriae and the breeding performance of a colonial seabird, king penguin Aptenodytes patagonicus. J Avian Biol. 2003;34: 30–34. 10.1034/j.1600-048X.2003.02916.x

[17] Benoit JB, Yoder JA, Lopez-Martinez G, Elnitsky MA, Lee RE, Denlinger DL. Habitat requirements of the seabird tick, Ixodes uriae (Acari: Ixodidae), from the Antarctic Peninsula in relation to water balance characteristics of eggs, nonfed and engorged stages. J Comp Physiol B, Biochem Syst Environ Physiol. 2007;177: 205–215. 10.1007/s00360-006-0122-7 17115223

[18] Muzaffar SB, Jones IL. Activity periods and questing behavior of the seabird tick Ixodes uriae (Acari: Ixodidae) on Gull Island, Newfoundland: the role of puffin chicks. J Parasitol. 2007;93: 258–264. 10.1645/GE-877R1.1 17539407

[19] Frenot Y, de Oliveira E, Gauthier-Clerc M, Deunff J, Bellido A, Vernon P. Life cycle of the tick Ixodes uriae in penguin colonies: relationships with host breeding activity. Int J Parasitol. 2001;31: 1040–1047. 10.1016/s0020-7519(01)00232-6 11429167

[20] Sonenshine DE. Biology of ticks. New York: Oxford University Press; 1991.

[21] Lee RE, Baust JG. Cold-hardiness in the Antarctic tick, Ixodes uriae. Physiological Zoology. 1987;60: 499–506. 10.1086/physzool.60.4.30157912

[22] Olsen B, Jaenson TG, Noppa L, Bunikis J, Bergström S. A Lyme borreliosis cycle in seabirds and Ixodes uriae ticks. Nature. 1993;362: 340–342. 10.1038/362340a0 8455718

[23] Gylfe Å, Olsen B, Strasevicius D, Marti Ras N, Weihe P, Noppa L, et al Isolation of Lyme disease Borrelia from puffins (Fratercula arctica) and seabird ticks (Ixodes uriae) on the Faeroe Islands. J Clin Microbiol. 1999;37: 890–896. 10.1128/JCM.37.4.890-896.1999 10074497 PMC84640

[24] St George TD, Doherty RL, Carley JG, Filippich C, Brescia A, Casals J, et al The isolation of arboviruses including a new flavivirus and a new Bunyavirus from Ixodes (Ceratixodes) uriae (Ixodoidea: Ixodidae) collected at Macquarie Island, Australia, 1975–1979. Am J Trop Med Hyg. 1985;34: 406–412. 10.4269/ajtmh.1985.34.406 2984951

[25] Major L, Linn ML, Slade RW, Schroder WA, Hyatt AD, Gardner J, et al Ticks associated with macquarie island penguins carry arboviruses from four genera. PLoS ONE. 2009;4: e4375 10.1371/journal.pone.0004375 19194498 PMC2632750

[26] Dietrich M, Kempf F, Boulinier T, McCoy KD. Tracing the colonization and diversification of the worldwide seabird ectoparasite Ixodes uriae. Mol Ecol. 2014;23: 3292–3305. 10.1111/mec.12815 24888342

[27] Wille M, Harvey E, Shi M, Gonzalez-Acuña D, Holmes EC, Hurt AC. Sustained virome diversity in Antarctic penguins and their ticks: geographical connectedness and no evidence for low pathogen pressure. Microbiology; 2019 12 10.1101/2019.12.11.873513

[28] Doherty R, Carley J, Murray M, Main AJ Jr, Kay B, Domrow R. Isolation of arboviruses (Kemerovo group, Sakhalin group) from Ixodes uriae collected at Macquarie Island, Southern Ocean. Am J Trop Med Hyg. 1975;24: 521–526. 10.4269/ajtmh.1975.24.521 50749

[29] Main AJ, Downs WG, Shope RE, Wallis RC. Avalon and Clo Mor: two new Sakhalin group viruses from the North Atlantic. J Med Entomol. 1976;13: 309–315. 10.1093/jmedent/13.3.309 1011234

[30] L’vov DK, Timopheeva AA, Gromashevski VL, Tsyrkin YuM, Veselovskaya OV, Gostinshchikova GV, et al “Okhotskiy” virus, a new arbovirus of the Kemerovo group isolated from ixodes (Ceratixodes) putus Pick.-Camb. 1878 in the Far East. Archiv für die gesamte Virusforschung. 1973;41: 160–164. 10.1007/BF01252760 4727777

[31] L’vov DK, Al’khovskiĭ SV, Shchelkanov MI, Shchetinin AM, Deriabin PG, Gitel’man AK, et al Molecular-genetic characterization of the Okhotskiy virus (OKHV) and Aniva virus (ANIV) (Orbivirus, Reoviridae) isolated from the ticks Ixodes (Ceratixodes) uriae White, 1852 in high latitudes of the Northern Eurasia. Vopr Virusol. 2014;59: 22–28. 25069281

[32] McCoy KD, Chapuis E, Tirard C, Boulinier T, Michalakis Y, Bohec CL, et al Recurrent evolution of host-specialized races in a globally distributed parasite. Proc Biol Sci. 2005;272: 2389–2395. 10.1098/rspb.2005.3230 16243689 PMC1559958

[33] Kempf F, Boulinier T, De Meeûs T, Arnathau C, McCoy KD. Recent evolution of host-associated divergence in the seabird tick Ixodes uriae. Mol Ecol. 2009;18: 4450–4462. 10.1111/j.1365-294X.2009.04356.x 19793353

[34] Safonova MV, Shchelkanov MY, Khafizov K, Matsvay AD, Ayginin AA, Dolgova AS, et al Sequencing and genetic characterization of two strains Paramushir virus obtained from the Tyuleniy Island in the Okhotsk Sea (2015). Ticks Tick Borne Dis. 2019;10: 269–279. 10.1016/j.ttbdis.2018.11.004 30448254

[35] Quillien MC, Monnat JY, Le Lay G, Le Goff F, Hardy E, Chastel C. Avalon virus, Sakhalin group (Nairovirus, Bunyaviridae) from the seabird tick Ixodes (Ceratixodes) uriae White 1852 in France. Acta Virol. 1986;30: 418–427. 2878589

[36] Olsen B, Munster VJ, Wallensten A, Waldenström J, Osterhaus ADME, Fouchier RAM. Global patterns of influenza a virus in wild birds. Science. 2006;312: 384–388. 10.1126/science.1122438 16627734

[37] Harvey E, Rose K, Eden J-S, Lo N, Abeyasuriya T, Shi M, et al Extensive Diversity of RNA Viruses in Australian Ticks. J Virol. 2019;93 10.1128/JVI.01358-18 30404810 PMC6340049

[38] Pettersson JH-O, Shi M, Bohlin J, Eldholm V, Brynildsrud OB, Paulsen KM, et al Characterizing the virome of Ixodes ricinus ticks from northern Europe. Sci Rep. 2017;7: 10870 10.1038/s41598-017-11439-y 28883464 PMC5589870

[39] Nakao R, Matsuno K, Qiu Y, Maruyama J, Eguchi N, Nao N, et al Putative RNA viral sequences detected in an Ixodes scapularis-derived cell line. Ticks Tick Borne Dis. 2017;8: 103–111. 10.1016/j.ttbdis.2016.10.005 27769656

[40] Shi M, Lin X-D, Tian J-H, Chen L-J, Chen X, Li C-X, et al Redefining the invertebrate RNA virosphere. Nature. 2016;540: 539–543. 10.1038/nature20167 27880757

[41] Cholleti H, Hayer J, Mulandane FC, Falk K, Fafetine J, Berg M, et al Viral metagenomics reveals the presence of highly divergent quaranjavirus in Rhipicephalus ticks from Mozambique. Infect Ecol Epidemiol. 2018;8: 1478585 10.1080/20008686.2018.1478585 29868166 PMC5974704

[42] Briese T, Chowdhary R, Travassos da Rosa A, Hutchison SK, Popov V, Street C, et al Upolu virus and Aransas Bay virus, two presumptive bunyaviruses, are novel members of the family Orthomyxoviridae. J Virol. 2014;88: 5298–5309. 10.1128/JVI.03391-13 24574415 PMC4019087

[43] Bussetti AV, Palacios G, Travassos da Rosa A, Savji N, Jain K, Guzman H, et al Genomic and antigenic characterization of Jos virus. J Gen Virol. 2012;93: 293–298. 10.1099/vir.0.035121-0 21994326 PMC3352346

[44] Leahy MB, Dessens JT, Weber F, Kochs G, Nuttall PA. The fourth genus in the Orthomyxoviridae: sequence analyses of two Thogoto virus polymerase proteins and comparison with influenza viruses. Virus Res. 1997;50: 215–224. 10.1016/s0168-1702(97)00072-5 9282786

[45] Bennett AJ, Bushmaker T, Cameron K, Ondzie A, Niama FR, Parra H-J, et al Diverse RNA viruses of arthropod origin in the blood of fruit bats suggest a link between bat and arthropod viromes. Virology. 2019;528: 64–72. 10.1016/j.virol.2018.12.009 30576861 PMC6401223

[46] Charrel RN, Attoui H, Butenko AM, Clegg JC, Deubel V, Frolova TV, et al Tick-borne virus diseases of human interest in Europe. Clin Microbiol Infect. 2004;10: 1040–1055. 10.1111/j.1469-0691.2004.01022.x 15606630

[47] Gritsun TS, Nuttall PA, Gould EA. Tick-borne flaviviruses. Adv Virus Res. 2003;61: 317–371. 10.1016/s0065-3527(03)61008-0 14714436

[48] Hubálek Z, Rudolf I. Tick-borne viruses in Europe. Parasitol Res. 2012;111: 9–36. 10.1007/s00436-012-2910-1 22526290

[49] Pettersson JH-O, Golovljova I, Vene S, Jaenson TG. Prevalence of tick-borne encephalitis virus in *Ixodes ricinus* ticks in northern Europe with particular reference to Southern Sweden. Parasites & Vectors. 2014;7: 102 10.1186/1756-3305-7-102 24618209 PMC4007564

[50] Medlock JM, Hansford KM, Bormane A, Derdakova M, Estrada-Peña A, George J-C, et al Driving forces for changes in geographical distribution of *Ixodes ricinus* ticks in Europe. Parasit Vectors. 2013;6: 1 10.1186/1756-3305-6-1 23281838 PMC3549795

[51] Jaenson TG, Hjertqvist M, Bergstrom T, Lundkvist A. Why is tick-borne encephalitis increasing? A review of the key factors causing the increasing incidence of human TBE in Sweden. Parasit Vectors. 2012;5: 184 10.1186/1756-3305-5-184 22937961 PMC3439267

[52] Jongejan F, Uilenberg G. The global importance of ticks. Parasitology. 2004;129 Suppl: S3–14. 10.1017/s0031182004005967 15938502

[53] Labuda M, Nuttall PA. Tick-borne viruses. Parasitology. 2004;129 Suppl: S221–245. 10.1017/s0031182004005220 15938513

[54] Parola P, Paddock CD, Socolovschi C, Labruna MB, Mediannikov O, Kernif T, et al Update on tick-borne rickettsioses around the world: a geographic approach. Clin Microbiol Rev. 2013;26: 657–702. 10.1128/CMR.00032-13 24092850 PMC3811236

[55] Tokarz R, Williams SH, Sameroff S, Sanchez Leon M, Jain K, Lipkin WI. Virome analysis of *Amblyomma americanum*, *Dermacentor variabilis*, and *Ixodes scapularis* ticks reveals novel highly divergent vertebrate and invertebrate viruses. Journal of Virology. 2014;88: 11480–11492. 10.1128/JVI.01858-14 25056893 PMC4178814

[56] Humphery-Smith I, Cybinski DH, Byrnes KA, St George TD. Seroepidemiology of arboviruses among seabirds and island residents of the Great Barrier Reef and Coral Sea. Epidemiol Infect. 1991;107: 435–440. 10.1017/s0950268800049086 1657626 PMC2272077

[57] Lʹvov DK, Shchelkanov MY, Alkhovsky SV, Deryabin PG. Zoonotic viruses of Northern Eurasia: taxonomy and ecology. Amsterdam: Elsevier/Academic Press; 2015.

[58] Grard G, Moureau G, Charrel RN, Lemasson J-J, Gonzalez J-P, Gallian P, et al Genetic characterization of tick-borne flaviviruses: New insights into evolution, pathogenetic determinants and taxonomy. Virology. 2007;361: 80–92. 10.1016/j.virol.2006.09.015 17169393

[59] Åkesson S, Ilieva M, Karagicheva J, Rakhimberdiev E, Tomotani B, Helm B. Timing avian long-distance migration: from internal clock mechanisms to global flights. Philos Trans R Soc Lond, B, Biol Sci. 2017;372 10.1098/rstb.2016.0252 28993496 PMC5647279

[60] Brooke M. Albatrosses and petrels across the world. Oxford; New York: Oxford University Press; 2004.

[61] Gaston AJ, Jones IL. The auks: Alcidae. Oxford; New York: Oxford University Press; 1998.

[62] Frederiksen M, Moe B, Daunt F, Phillips RA, Barrett RT, Bogdanova MI, et al Multicolony tracking reveals the winter distribution of a pelagic seabird on an ocean basin scale. Divers Distrib. 2012;18: 530–542.

[63] Egevang C, Stenhouse IJ, Phillips RA, Petersen A, Fox JW, Silk JRD. Tracking of Arctic terns Sterna paradisaea reveals longest animal migration. Proc Natl Acad Sci USA. 2010;107: 2078–2081. 10.1073/pnas.0909493107 20080662 PMC2836663

[64] Carey MJ, Phillips RA, Silk JR, Shaffer SA. Trans-equatorial migration of Short-tailed Shearwaters revealed by geolocators. Emu-Austral Ornithology. 2014;114: 352–359.

[65] Shaffer SA, Tremblay Y, Weimerskirch H, Scott D, Thompson DR, Sagar PM, et al Migratory shearwaters integrate oceanic resources across the Pacific Ocean in an endless summer. Proc Natl Acad Sci USA. 2006;103: 12799–12802. 10.1073/pnas.0603715103 16908846 PMC1568927

[66] Murray M, Vestjens W. Studies on the ectoparasites of seals and penguins. III. The distribution of the tick Ixodes uriae White and the flea Parapsyllus magellanicus heardi de Meillon on Macquarie Island. Aust J Zool. 1967;15: 715–725.

[67] Randolph SE, Gern L, Nuttall PA. Co-feeding ticks: Epidemiological significance for tick-borne pathogen transmission. Parasitol Today (Regul Ed). 1996;12: 472–479. 15275266 10.1016/s0169-4758(96)10072-7

[68] Randolph SE. Transmission of tick-borne pathogens between co-feeding ticks: Milan Labuda’s enduring paradigm. Ticks Tick Borne Dis. 2011;2: 179–182. 10.1016/j.ttbdis.2011.07.004 22108009

[69] McCoy KD, Léger E, Dietrich M. Host specialization in ticks and transmission of tick-borne diseases: a review. Front Cell Infect Microbiol. 2013;3: 57 10.3389/fcimb.2013.00057 24109592 PMC3790072

[70] Jones LD, Davies CR, Steele GM, Nuttall PA. A novel mode of arbovirus transmission involving a nonviremic host. Science. 1987;237: 775–777. 10.1126/science.3616608 3616608

[71] Nuttall PA, Labuda M. Dynamics of infection in tick vectors and at the tick-host interface. Adv Virus Res. 2003;60: 233–272. 10.1016/s0065-3527(03)60007-2 14689696

[72] Petney TN, Pfäffle MP. Ixodes uriae White, 1852 (Figs. 38–40) In: Estrada-Peña A, Mihalca AD, Petney TN, editors. Ticks of Europe and North Africa. Cham: Springer International Publishing; 2017 pp. 115–119. 10.1007/978-3-319-63760-0_23

[73] Pettersson JH-O, Shi M, Eden J-S, Holmes EC, Hesson JC. Meta-transcriptomic comparison of the RNA viromes of the mosquito vectors *Culex pipiens* and *Culex torrentium* in northern Europe. Viruses. 2019;11 10.3390/v11111033 31698792 PMC6893722

[74] Bolger AM, Lohse M, Usadel B. Trimmomatic: a flexible trimmer for Illumina sequence data. Bioinformatics. 2014;30: 2114–2120. 10.1093/bioinformatics/btu170 24695404 PMC4103590

[75] Haas BJ, Papanicolaou A, Yassour M, Grabherr M, Blood PD, Bowden J, et al De novo transcript sequence reconstruction from RNA-seq using the Trinity platform for reference generation and analysis. Nat Prot. 2013;8: 1494–1512. 10.1038/nprot.2013.084 23845962 PMC3875132

[76] Camacho C, Coulouris G, Avagyan V, Ma N, Papadopoulos J, Bealer K, et al BLAST+: architecture and applications. BMC Bioinformatics. 2009;10: 421 10.1186/1471-2105-10-421 20003500 PMC2803857

[77] Buchfink B, Xie C, Huson DH. Fast and sensitive protein alignment using DIAMOND. Nat Methods. 2015;12: 59–60. 10.1038/nmeth.3176 25402007

[78] Langmead B, Salzberg SL. Fast gapped-read alignment with Bowtie 2. Nat Meth. 2012;9: 357–359. 10.1038/nmeth.1923 22388286 PMC3322381

[79] Katoh K, Standley DM. MAFFT multiple sequence alignment software version 7: Improvements in performance and usability. Mol Biol Evol. 2013;30: 772–780. 10.1093/molbev/mst010 23329690 PMC3603318

[80] Capella-Gutierrez S, Silla-Martinez JM, Gabaldon T. trimAl: a tool for automated alignment trimming in large-scale phylogenetic analyses. Bioinformatics. 2009;25: 1972–1973. 10.1093/bioinformatics/btp348 19505945 PMC2712344

[81] Kalyaanamoorthy S, Minh BQ, Wong TKF, von Haeseler A, Jermiin LS. ModelFinder: fast model selection for accurate phylogenetic estimates. Nat Methods. 2017;14: 587–589. 10.1038/nmeth.4285 28481363 PMC5453245

[82] Nguyen L-T, Schmidt HA, von Haeseler A, Minh BQ. IQ-TREE: a fast and effective stochastic algorithm for estimating maximum-likelihood phylogenies. Mol Biol Evol. 2015;32: 268–274. 10.1093/molbev/msu300 25371430 PMC4271533

[83] Larsson A. AliView: a fast and lightweight alignment viewer and editor for large datasets. Bioinformatics. 2014;30: 3276–3278. 10.1093/bioinformatics/btu531 25095880 PMC4221126

[84] Kumar S, Stecher G, Li M, Knyaz C, Tamura K. MEGA X: Molecular Evolutionary Genetics Analysis across Computing Platforms. Mol Biol Evol. 2018;35: 1547–1549. 10.1093/molbev/msy096 29722887 PMC5967553

