# Supplementary material for: Circumpolar diversification of the Ixodes uriae tick virome
Source: PLoS Pathog. 2020 Aug 3;16(8):e1008759. doi: 10.1371/journal.ppat.1008759 (PMC7425989; doi:10.1371/journal.ppat.1008759)

Supplementary figure S1. Phylogenetic reconstruction of *Ixodes uriae* based on mitochondrial sequences. Sequences from the southern circumpolar region are coloured red. Sequences from the northern circumpolar region are coloured in blue. All branches are scaled according to the number of nucleotide substitutions per site. Values on indicate bootstrap support. Only values above 80 are indicated. The tree is rooted with *Ixodes holocyclus*.

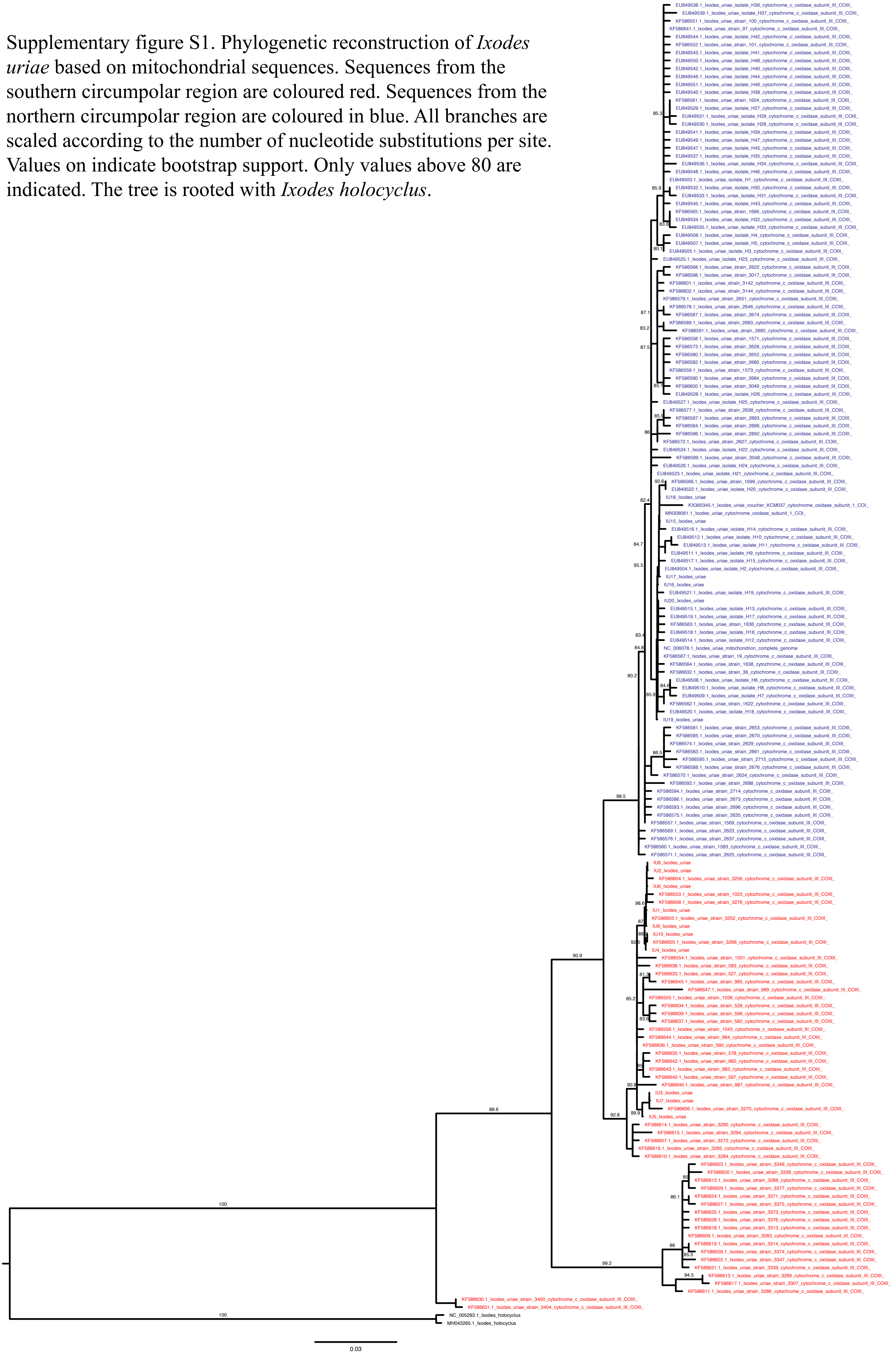

Supplement: S1 Fig — (PDF) [file ppat.1008759.s003.pdf]
